# To the Editors of the Medical and Physical Journal

**Published:** 1805-09-01

**Authors:** Edw. Leese

**Affiliations:** East Street, Manchester Square


### *To the Editors of the Medical and Phyſical Journal.*

GENTLEMEN,

THINKING the following account of the second appearance of small-pox in the same person, interesting; and that it may be usefully conveyed to the public, through the medium of your very useful and widely circulated Journal, I have sent it for insertion.

*East Street, Manchester Square,* I am, &c.

*August* 18, 1805. EDW. LEESE.

---

Mrs. PIDGEON, now living as housekeeper with Mr. Jarnet, of David Street, the corner of East Street, Manchester Square, informs me, that her late husband, when about fifteen years of age, then residing as a gardener with Capt. Markall, of Honiton, in Devonshire, was inoculated for the small-pox by Mr. Buller, a surgeon of that place; that his arm inflamed very much; and that it was succeeded by a thick eruption of pustules all over his body; his feet were so full of them, that he was unable to stand. He was for some time confined; and so very ill, that Dr. Robinson, of Honiton, was requested to see him. Dr. Robinson and Mr. Buller continued to attend him till his recovery, without expressing any doubt of the nature of the disease.

Some years after, a lad in the same family had the small-pox; during the progress of which, he slept with him, and received no inconvenience.

When he was about twenty-eight years of age, his own children had the small-pox; of which one died. He lived and slept with them, and at length was taken so ill, as to be obliged to leave his work; complaining of sickness, head-ach, and severe pains in his back. The medical gentleman attending the children was sent to; who observed, that if he had not already had the small-pox, he should think he was then sickening for it. After an illness of two or three days, a full eruption of the confluent small-pox made its appearance; of which he died, on the eleventh day.

